# Apocrine Hidrocystoma of Foreskin: A Rare Benign Cystic Lesion

**DOI:** 10.7759/cureus.107284

**Published:** 2026-04-18

**Authors:** Lin Aung Han, Laura Potter, Mallikarjun Bardapure

**Affiliations:** 1 Urology, Southmead Hospital, Bristol, GBR; 2 Pathology, Royal Shrewsbury Hospital, Shrewsbury, GBR; 3 Urology, Royal Shrewsbury Hospital, Shrewsbury, GBR

**Keywords:** apocrine hidrocystoma, benign cystic tumour, circumcision, foreskin, penis

## Abstract

Apocrine hidrocystoma is a benign cystic tumour arising from apocrine sweat glands. Its occurrence on the penile foreskin is exceedingly rare, representing an uncommon lesion of the external genitalia. A review of the literature suggests that only a limited number of cases -- approximately 13 involving external genitalia -- have been reported worldwide.

We present the case of a 50-year-old gentleman who was referred to the urology service with a gradually enlarging, multiloculated, non-proliferative cyst arising from the foreskin. Patient underwent excisional biopsy with complete circumcision under local anaesthesia. Histopathology examination confirmed the diagnosis of apocrine hidrocystoma.

Apocrine hidrocystomas are mostly located in the head and neck region, particularly the periorbital area, external auditory meatus, and axilla, with genital involvement being exceptionally uncommon. Complete surgical excision is both diagnostic and curative with a low risk of recurrence.

Reports of apocrine hidrocystoma involving the foreskin remain scarce in the literature. To our knowledge, this represents the first reported case from Royal Shrewsbury Hospital.

## Introduction

The pathogenesis of apocrine hidrocystoma remains incompletely understood. It is thought to arise from cystic dilatation of apocrine secretory coils. Historically, apocrine hidrocystoma and apocrine cystadenoma have been used interchangeably, leading to confusion about the nature of the lesions. However, they are now considered distinct entities. Apocrine hidrocystoma has been shown as a retention-type cystic dilation of the eccrine sweat duct. Histologically, the key difference lies in the absence of a fibrous stromal core in apocrine hidrocystoma [1].

Clinically, apocrine hidrocystomas typically present as solitary cystic lesions that may be unilocular (approximately 70%) or multilocular. They are usually translucent, dome-shaped cysts with colour varying from blue, brown, or black to skin colour. Although these lesions can occur across a wide age range, they are more commonly observed in adults aged 30 to 70 years [2]. There is no clear gender predilection.

These lesions are most frequently found in the regions rich in apocrine glands, such as the head and neck -- particularly the periorbital area -- as well as the axilla. In contrast, involvement of the genitalia is exceedingly rare. When present, lesions commonly arise on the foreskin, followed by the penile shaft [3].

## Case presentation

Case history and presentation

A 50-year-old gentleman was seen and referred by a GP for the finding of a growth on the penis. Over the last 18 months, it has gradually increased in size. Apart from that, there was no notice of pain, bleeding, urinary symptoms, or pruritus. His main concerns were the spraying of urine and painful sexual intercourse because of the swelling. His past medical and surgical history was insignificant. There was no history of trauma or vigorous sexual activity.

Upon examination, a 1 x 1 cm cystic lesion was noted at the frenulum of the foreskin, which was smooth and not tender. Skin over the cyst was also normal. Another tiny cyst was noted at 6 o’clock of the external meatus. Apart from those, his external genitalia was unremarkable. There was no palpable inguinal lymph node.

Methods (treatment and histopathology)

Because of painful sexual intercourse, he consented to circumcision and biopsy of the cyst, which was performed under local anaesthesia. The operation was a routine dorsal slit and bipolar excision of the foreskin, including the frenular cyst.

Result

**Figure 1:**
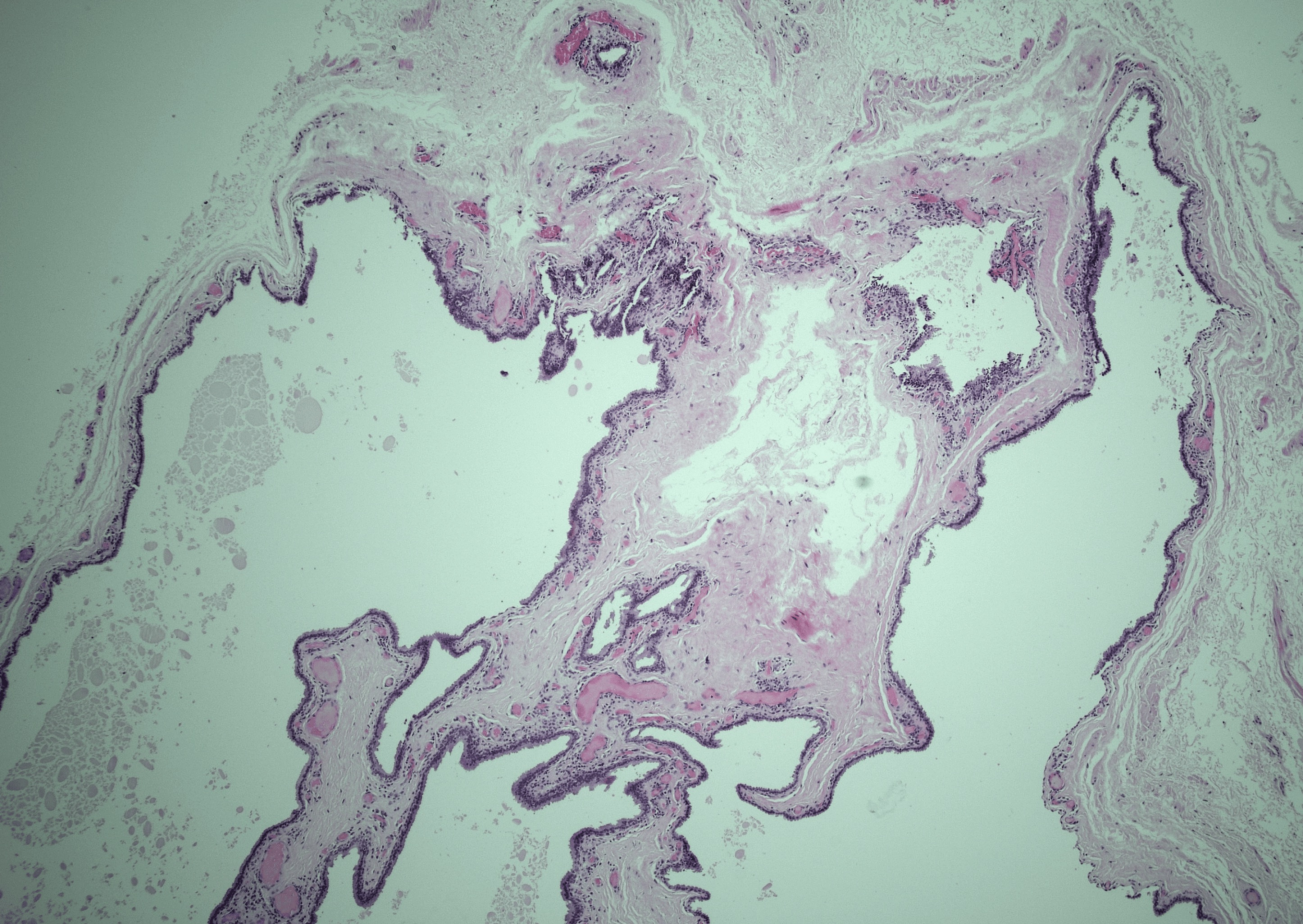
Histopathological feature of apocrine hidrocystoma (H&E, x4) showing large unilocular spaces in dermis

**Figure 2:**
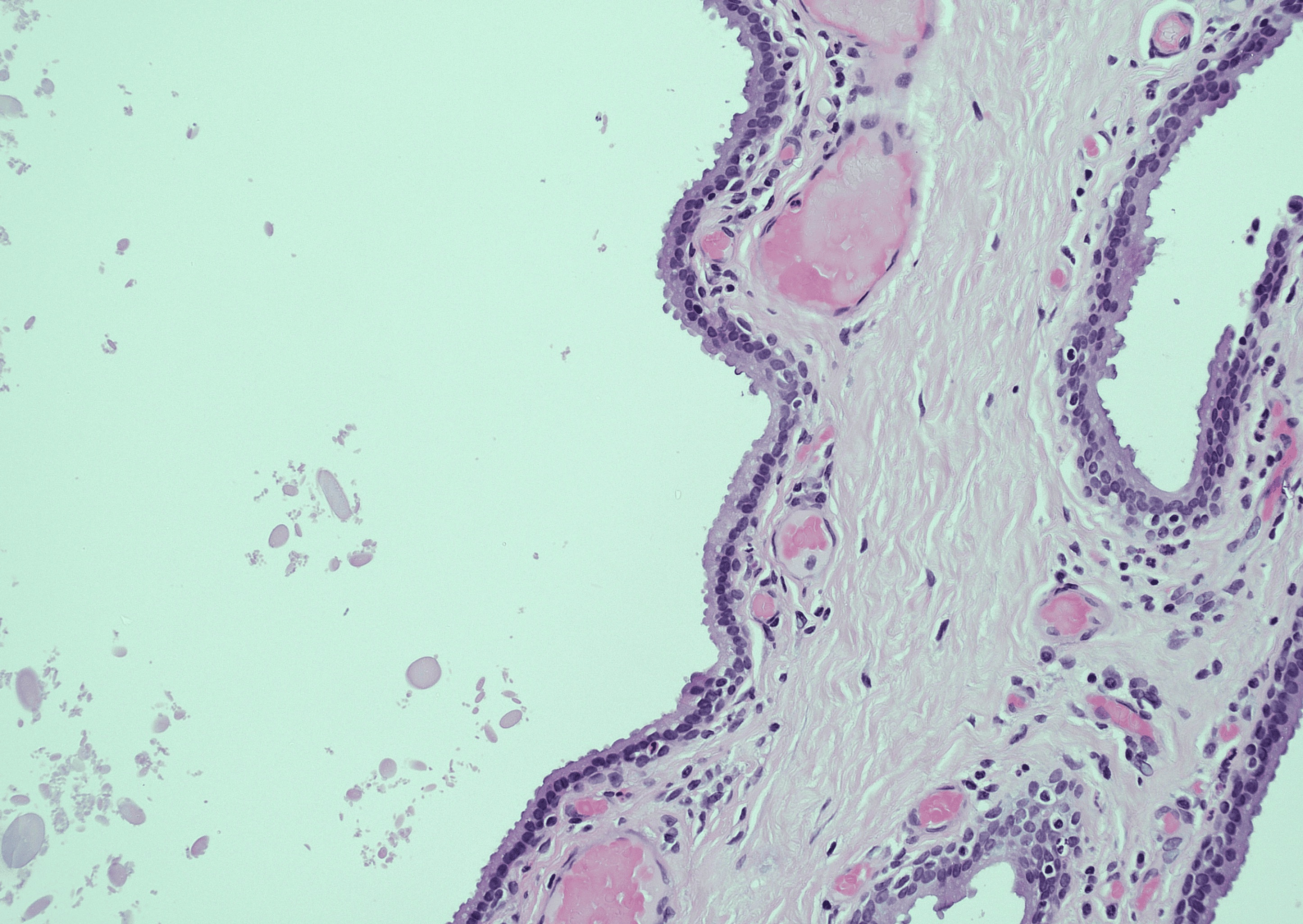
Histopathological feature of apocrine hidrocystoma (H&E, x20) showing large cystic structures in dermis lined by double layers of epithelial cells

Histological evaluation found a couple of large unilocular spaces in the dermis lined by a double layer of epithelial cells. Decapitation secretion was observed in some luminal cells, and no evidence of atypia or malignancy was noted. The histological features were consistent with an apocrine hidrocystoma (Figures 1, 2).

He had a follow-up via telephone clinic, where he was updated about the results. Fortunately, he seemed to have no issues with wound healing, and his primary symptom of painful sexual intercourse appeared to be resolved after the surgery.

## Discussion

Early descriptions of apocrine hidrocystoma were provided by Mehregan, who characterised it as a benign nevoid tumour representing an adenomatous cystic proliferation of apocrine glands. As a result, the lesion was historically referred to as apocrine cystadenoma. By the histological architecture, Mehregan also noted that some lesions demonstrate papillomatous hyperplasia of the cyst wall projecting into the cystic lumen, indicating variability in histological architecture [4].

Subsequently, Smith and Chernosky analysed 50 cases of apocrine lesions and observed that approximately half exhibited papillary projections into the cyst cavity, while the remainder lacked such features. Based on these findings, they proposed a distinction between the two entities: lesions without papillary projections should be classified as apocrine hidrocystomas, analogous to retention cysts of eccrine origin, whereas those with prominent papillary proliferations should be termed apocrine cystadenomas [5]. 

Histopathologically, these cysts are mainly composed of an inner layer of single- or double-secretory columnar epithelium with decapitation secretion, lying above an outer myoepithelial cell layer. Immunohistochemical study revealed that outer myoepithelial cells are positive for alpha-smooth muscle actin and p63, and inner epithelium is positive for CK7 and CK18 [6].

The principal differential diagnosis is eccrine hidrocystoma, which typically presents as smaller lesions that may fluctuate in size with environmental temperature, often enlarging during warmer conditions and regressing in cooler climates [7]. Immunohistochemical staining can aid in differentiation, as markers positive in apocrine hidrocystoma are generally negative in eccrine hidrocystoma. Other important differential diagnoses include epidermal inclusion cyst, median raphe cyst, and acquired lymphangioma.

Definitive management is surgical excision, which may be performed in the form of circumcision when the lesion involves the foreskin. Complete excision is both diagnostic and curative. Given the benign nature of apocrine hidrocystoma, wide surgical margins are not required. Recurrence has not been reported in the literature, although it remains theoretically possible [2]. To date, there is no evidence of malignant transformation associated with this lesion.

## Conclusions

We report a rare case of apocrine hidrocystoma arising from the foreskin in a 50-year-old man, presenting as a gradually enlarging cystic lesion associated with discomfort during sexual intercourse. This case highlights the importance of considering uncommon benign entities in the differential diagnosis of penile lesions, particularly when clinical features are non-specific. Accurate diagnosis relies on histopathological confirmation, as clinical assessment alone may be insufficient to distinguish it from other cystic conditions. Complete surgical excision, in this case achieved through circumcision, is both diagnostic and curative, with excellent outcomes. Increased awareness of this rare condition can help clinicians avoid misdiagnosis, provide appropriate management, and reassure patients regarding its benign nature.

## Appendices

This article was previously presented as an unmoderated e-poster at the 45th Congress of the Société Internationale d'Urologie, Edinburgh, on October 29-November 1, 2025.
